# Missense mutations in the *TP53* DNA-binding domain predict outcomes in patients with advanced oral cavity squamous cell carcinoma

**DOI:** 10.18632/oncotarget.9925

**Published:** 2016-06-08

**Authors:** Nina Lapke, Yen-Jung Lu, Chun-Ta Liao, Li-Yu Lee, Chien-Yu Lin, Hung-Ming Wang, Shu-Hang Ng, Shu-Jen Chen, Tzu-Chen Yen

**Affiliations:** ^1^ ACT Genomics, Taipei, Taiwan, ROC; ^2^ Department of Otorhinolaryngology, Head and Neck Surgery, Chang Gung Memorial Hospital and Chang Gung University, Taoyuan, Taiwan, ROC; ^3^ Department of Pathology, Chang Gung Memorial Hospital and Chang Gung University, Taoyuan, Taiwan, ROC; ^4^ Department of Radiation Oncology, Chang Gung Memorial Hospital and Chang Gung University, Taoyuan, Taiwan, ROC; ^5^ Department of Medical Oncology, Chang Gung Memorial Hospital and Chang Gung University, Taoyuan, Taiwan, ROC; ^6^ Department of Diagnostic Radiology, Chang Gung Memorial Hospital and Chang Gung University, Taoyuan, Taiwan, ROC; ^7^ Department of Nuclear Medicine and Molecular Imaging Center, Chang Gung Memorial Hospital and Chang Gung University, Taoyuan, Taiwan, ROC

**Keywords:** oral cavity squamous cell carcinoma, missense mutations, TP53 DNA-binding domain, biomarker, outcome

## Abstract

*TP53* mutations have been linked to reduced survival in patients with oral cavity squamous cell carcinoma (OSCC). However, the impact of different types of *TP53* mutations remains unclear. Here, we demonstrate that the carriage of missense mutations in the *TP53* DNA binding domain (DBD missense mutations) is associated with decreased disease-specific survival (DSS) compared with wild-type *TP53* (P=0.002) in a cohort of 345 OSCC patients. In contrast, DSS of patients bearing all of the remaining *TP53* mutations did not differ from that observed in wild-type *TP53* patients (P=0.955). Our classification method for *TP53* mutations was superior to previously reported approaches (disruptive, truncating, Evolutionary Action score, mutations in L2/L3/LSH) for distinguishing between low- and high-risk patients. When analyzed in combination with traditional clinicopathological factors, *TP53* DBD missense mutations were an independent prognostic factor for shorter DSS (P=0.014) alongside with advanced AJCC T- and N-classifications and the presence of extracapsular spread. A scoring system that included the four independent prognostic factors allowed a reliable patient stratification into distinct risk groups (high-risk patients, 16.2%). Our results demonstrate the usefulness of *TP53* DBD missense mutations combined with clinicopathological factors for improving the prognostic stratification of OSCC patients.

## INTRODUCTION

Approximately 300,000 new cases of oral cavity cancer are diagnosed each year, with this malignancy being responsible for 150,000 deaths annually (GLOBOCAN 2012, http://globocan.iarc.fr). The main risk factors for oral cavity cancer include cigarette smoking, alcohol drinking [1], and betel nut chewing [2], the latter being highly prevalent in Southeast Asia. Oral cavity squamous cell carcinoma (OSCC) accounts for more than 90% of all oral cavity malignancies. Unfortunately, 5-year survival rates of patients with advanced OSCC remain poor [3].

*TP53* is the most commonly mutated gene in OSCC (60−80% of cases) [4, 5]. Although OSCC patients carrying *TP53* mutations have reduced survival compared to those with a wild-type status [6, 7], the prognostic impact of different types of *TP53* mutations remains poorly understood. A commonly used classification is based on a large study conducted by Poeta *et al.* [8] that enrolled 560 patients with squamous cell carcinoma of the head and neck (HNSCC). In this study, all of the mutations that introduced a stop codon or non-conservative mutations in specific DNA binding domains (DBDs) were defined as disruptive. Disruptive *TP53* mutations are associated with a significantly decreased survival [8, 9]. However, disruptive mutations include two biologically different subtypes, namely 1) truncating mutations associated with a loss of tumor suppressive activity, and 2) DBD missense mutations. Although truncating mutations have been associated with an unfavorable prognosis [9], further confirmation of these findings is necessary. DBD missense mutations can result in a gain-of-function, ultimately leading to cell invasion, migration, proliferation, and drug resistance [10]. Possible mechanisms leading to a gain-of-function include changes in DNA binding properties [11] and/or altered protein-protein interactions [12]. Notably, a study in breast cancer patients demonstrated that only *TP53* DBD missense mutations (and not other mutations) have an adverse prognostic impact [13]. It has been recently suggested that *TP53* missense mutations occurring in evolutionary conserved residues are likely to confer a gain-of-function, ultimately predicting poor treatment response and a shorter survival in HNSCC patients [14, 15]. Other studies focusing on mutations occurring in the DBD or DBD-defined regions (e.g., L2, L3 and LSH) [7, 16] have reported their adverse prognostic significance, although conflicting results exist [7, 9, 16, 17]. Such discrepancies can be ascribed to small sample sizes or sequencing areas limited to exons 5−8.

**Figure 1:**
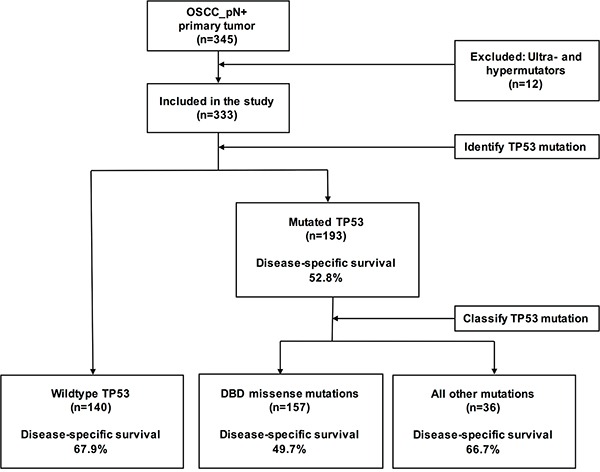
Flow of OSCC patients through the study and *TP53* mutation analysis

Starting from these premises, we designed the current study to shed more light on the prognostic impact of different *TP53* mutation types in a large cohort of 345 patients with advanced (AJCC stage III/IV) OSCC (Figure 1). Ultra-deep targeted sequencing (average sequencing depth > 2000×) of formalin-fixed paraffin-embedded (FFPE) tumor samples was performed for exons 2, 4−8, and 10. These regions covered all of the relevant hotspots for head and neck cancer identified in the TCGA HNSCC cohort. Working from the assumption that most DBD missense mutations can lead to a gain-of-function [10, 18], we categorized *TP53* mutations into two distinct categories, i.e., DBD missense mutations *versus* all other mutations. We then compared the predictive value of *TP53* DBD missense mutations *versus* other types of *TP53* mutations in terms of disease-specific survival (DSS). Furthermore, we combined *TP53* DBD missense mutations with traditional risk factors with the aim of identifying high-risk patients.

## RESULTS

### Patient characteristics

The general characteristics of the study patients are listed in Table 1. In line with previous methodology [19], ultra- and hypermutators (n = 12) were excluded. A total of 333 patients were eligible for the study. There was a clear preponderance of male subjects (94.0%, n=313). The median age was 48 years (range: 27−89 years) and the median follow-up time after surgery was 50 months. The distribution of known risk factors for OSCC was as follows: pre-operative smoking (90.4%, n=301), pre-operative betel nut chewing (81.4%, n=271), pre-operative alcohol drinking (71.2%, n=237), and HPV16/18 infections (12.6%, n=42; subjects with known HPV16/18 status: n=317). The tumor sites were as follows: buccal (38.1%, n=127), retromolar trigone (4.5%, n=15), lip (0.6%, n=2), tongue (37.5%, n=125), alveolar ridge (12.9%, n=43), hard palate (1.8%, n=6), and floor of mouth (4.5%, n=15). Extracapsular spread (ECS) was observed in 58.0% (n=193) of the study patients.

**Table 1:** General characteristics of the study patients (n = 333) according the *TP53* mutation status

Characteristics	Entire cohort		TP53 Wt		TP53 mutations		P value
	N	(%)	N	(%)	N	(%)	
**Entire study cohort**	333	(100.0%)	140	(42.0%)	193	(58.0%)	
**Sex**							0.249
Male	313	(94.0%)	129	(92.1%)	184	(95.3%)	
Female	20	(6.0%)	11	(7.9%)	9	(4.7%)	
**Age, years**							
Median	48		47		49		
Range	27	−89	29	−89	27	−83	
Mean ± SD	49.7	±11.0	49.5	±11.7	49.9	±10.6	
**Age (categorical)**							0.167
<65 years	295	(88.6%)	120	(85.7%)	175	(90.7%)	
≥65 years	38	(11.4%)	20	(14.3%)	18	(9.3%)	
**Cigarette smoking**							0.577
No	32	(9.6%)	15	(10.7%)	17	(8.8%)	
Yes	301	(90.4%)	125	(89.3%)	176	(91.2%)	
**Betel chewing**							0.318
No	62	(18.6%)	30	(21.4%)	32	(16.6%)	
Yes	271	(81.4%)	110	(78.6%)	161	(83.4%)	
**Alcohol drinking**							0.038
No	96	(28.8%)	49	(35.0%)	47	(24.4%)	
Yes	237	(71.2%)	91	(65.0%)	146	(75.6%)	
**HPV16/18 positive**							0.403
No	275	(82.6%)	111	(79.3%)	164	(85.0%)	
Yes	42	(12.6%)	20	(14.3%)	22	(11.4%)	
Unknown	16	(4.8%)	9	(6.4%)	7	(3.6%)	
**Tumor site**							0.290
Buccal	127	(38.1%)	61	(43.6%)	66	(34.2%)	
Retromolar Trigone	15	(4.5%)	3	(2.1%)	12	(6.2%)	
Lip	2	(0.6%)	1	(0.7%)	1	(0.5%)	
Tongue	125	(37.5%)	46	(32.9%)	79	(40.9%)	
Alveolar ridge	43	(12.9%)	21	(15.0%)	22	(11.4%)	
Hard palate	6	(1.8%)	2	(1.4%)	4	(2.1%)	
Mouth floor	15	(4.5%)	6	(4.3%)	9	(4.7%)	
**AJCC T-classification**							0.147
pT1-2	148	(44.4%)	69	(49.3%)	79	(40.9%)	
pT3-4	185	(55.6%)	71	(50.7%)	114	(59.1%)	
**AJCC N-classification**							0.297
pN1	119	(35.7%)	55	(39.3%)	64	(33.2%)	
pN2	214	(64.3%)	85	(60.7%)	129	(66.8%)	
**AJCC staging (overall)**							0.096
p-Stage III	82	(24.6%)	41	(29.3%)	41	(21.2%)	
p-Stage IV	251	(75.4%)	99	(70.7%)	152	(78.8%)	
**Extracapsular spread**							0.370
No	140	(42.0%)	63	(45.0%)	77	(39.9%)	
Yes	193	(58.0%)	77	(55.0%)	116	(60.1%)	
**Differentiation**							0.929
Well	60	(18.0%)	24	(17.1%)	36	(18.7%)	
Moderate	220	(66.1%)	93	(66.4%)	127	(65.8%)	
Poor	53	(15.9%)	23	(16.4%)	30	(15.5%)	
**Bone marrow invasion**							0.218
No	264	(79.3%)	116	(82.9%)	148	(76.7%)	
Yes	69	(20.7%)	24	(17.1%)	45	(23.3%)	
**Skin invasion**							0.716
No	299	(89.8%)	127	(90.7%)	172	(89.1%)	
Yes	34	(10.2%)	13	(9.3%)	21	(10.9%)	
**Perineural invasion**							1.000
No	162	(48.6%)	68	(48.6%)	94	(48.7%)	
Yes	171	(51.4%)	72	(51.4%)	99	(51.3%)	
**Vascular invasion**							0.812
No	315	(94.6%)	132	(94.3%)	183	(94.8%)	
Yes	18	(5.4%)	8	(5.7%)	10	(5.2%)	
**Lymphatic invasion**							0.619
No	291	(87.4%)	124	(88.6%)	167	(86.5%)	
Yes	42	(12.6%)	16	(11.4%)	26	(13.5%)	
**Margin status**							0.017
< 5 mm	41	(12.3%)	10	(7.1%)	31	(16.1%)	
≥ 5 mm	288	(86.5%)	129	(92.1%)	159	(82.4%)	
Unknown	4	(1.2%)	1	(0.7%)	3	(1.6%)	
**Tumor depth**							0.127
< 10 mm	111	(33.3%)	53	(37.9%)	58	(30.1%)	
≥ 10 mm	221	(66.4%)	86	(61.4%)	135	(69.9%)	
Unknown	1	(0.3%)	1	(0.7%)	0	(0.0%)	
**Local recurrence**							0.781
No	267	(80.2%)	111	(79.3%)	156	(80.8%)	
Yes	66	(19.8%)	29	(20.7%)	37	(19.2%)	
**Neck recurrence**							0.795
No	254	(76.3%)	108	(77.1%)	146	(75.6%)	
Yes	79	(23.7%)	32	(22.9%)	47	(24.4%)	
**Distant metastasis**							0.002
No	252	(75.7%)	118	(84.3%)	134	(69.4%)	
Yes	81	(24.3%)	22	(15.7%)	59	(30.6%)	
**Level IV/V metastases**							0.400
No	308	(92.5%)	132	(94.3%)	176	(91.2%)	
Yes	25	(7.5%)	8	(5.7%)	17	(8.8%)	
**Second primary tumor**							0.257
No	270	(81.1%)	118	(84.3%)	152	(78.8%)	
Yes	63	(18.9%)	22	(15.7%)	41	(21.2%)	
**Relapse after complete treatment**							0.121
No	171	(51.4%)	79	(56.4%)	92	(47.7%)	
Yes	162	(48.6%)	61	(43.6%)	101	(52.3%)	
**OSCC-related death**							0.007
No	197	(59.2%)	95	(67.9%)	102	(52.8%)	
Yes	136	(40.8%)	45	(32.1%)	91	(47.2%)	
**Death from any cause**							0.002
No	135	(40.5%)	71	(50.7%)	64	(33.2%)	
Yes	198	(59.5%)	69	(49.3%)	129	(66.8%)	

Categorical data were compared with the Fisher's exact test or the χ^2^ test, as appropriate.

### *TP53* mutations

*TP53* mutations were observed in 58.0% (n=193) of the study patients, with 228 mutations being identified (because two patients harbored three mutations and 31 patients two mutations). The following types of *TP53* mutations were observed: missense (78.1%, n=178), stop-gain (15.4%, n=35), splice site (3.5%, n=8), frameshift deletions (1.8%, n=4), and inframe deletions (1.3%, n=3). A total of 68 different mutated amino acids were identified. A list of all of the observed mutations is provided in the Supplementary Tables 1 and 2. The general characteristics of the study participants are reported in Supplementary Table 3. In total, 81.3% (n=157) of patients with mutated *TP53* harbored a *TP53* DBD missense mutation. Most (98.3%, n=175) missense mutations occurred in the DBD, with high frequencies being observed for the hotspots R273 (13.5%, n=24), R248 (11.2%, n=20), and R175 (9.6%, n=17).

### Survival in OSCC patients in relation to the presence of *TP53* mutations

**Figure 2:**
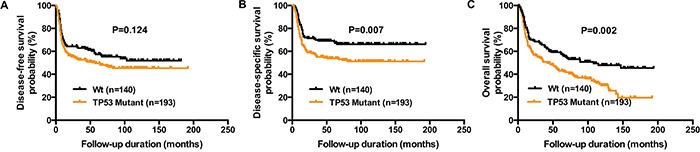
Reduced DFS, DSS, and OS in OSCC patients harboring *TP53* mutations Kaplan-Meier plots depict DFS **panel A.**, DSS **panel B.**, and OS **panel C.** of 333 OSCC patients according to the presence of any *TP53* mutation versus wild-type *TP53*. P values were calculated with the log-rank test.

We then compared the characteristics of patients harboring a *TP53* mutation (regardless of the mutation type) with those having wild-type *TP53* (Table 1). *TP53* mutations were associated with alcohol drinking, a margin status of less than 5 mm and a higher rate of distant metastases (P=0.038, P=0.017, and P=0.002, respectively). DFS was not significantly lower in patients with mutated *TP53* compared with wild-type *TP53* (HR, 1.28; 95% CI, 0.94−1.75; P=0.124; Figure 2A). However, DSS and OS were found to differ significantly between the two groups (DSS: HR, 1.62; 95% CI, 1.15−2.25; P=0.007, OS: HR, 1.59; 95% CI, 1.20−2.09; P=0.002; Figure 2B and 2C).

### *TP53* DBD missense mutations allow an optimal stratification of *TP53*-mutant patients

**Figure 3:**
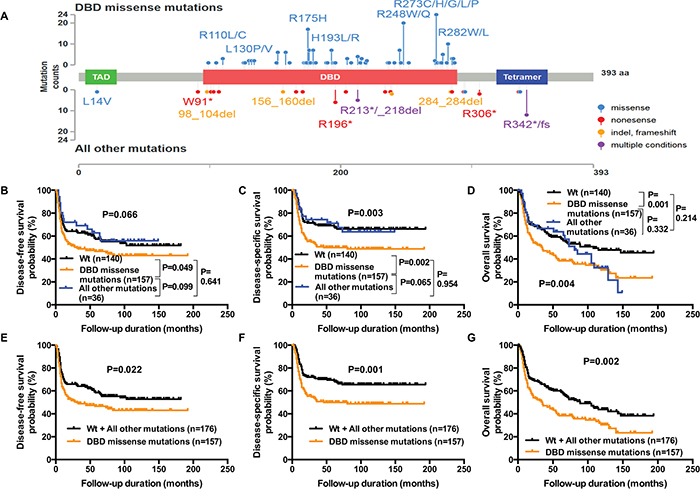
Reduced DFS, DSS, and OS in OSCC patients harboring *TP53* DBD missense mutations Missense *TP53* mutations in the DNA binding domain (residues 95−289) were considered as “*TP53* DBD missense mutations”, whereas all of the remaining mutations were defined as “all other mutations”. The distribution of *TP53* DBD missense mutations and all other mutations in the study patients (n=333) is shown in **panel A.** Kaplan-Meier plots depict DFS **panel B.**, DSS **panel C.**, and OS **panel D.** of patients with *TP53* DBD missense mutations *versus* all other mutations. Patients with all other mutations and wild-type *TP53* were then merged into a single group and compared with patients carrying *TP53* DBD missense mutations. After grouping, Kaplan-Meier plots of DFS **panel E.**, DSS **panel F.**, and OS **panel G.** were constructed. P values were calculated with the log-rank test.

Based on the assumption that *TP53* DBD missense mutations would lead to a gain-of-function, we hypothesized that this mutation subtype may lead to dismal outcomes. We therefore divided patients with *TP53* mutations into two subgroups (i.e., *TP53* DBD missense mutations *versus* all other mutations, Figure 3A). *TP53* DBD missense mutations were associated with a decreased DSS compared to wild-type *TP53* (HR, 1.78; 95% CI, 1.23−2.57; P=0.002; Table 2). However, DSS of patients with all other mutations was comparable to patients with wild-type *TP53* (HR, 1.02; 95% CI, 0.54−1.93; P=0.955).

**Table 2:** *TP53* mutations and DSS according to different *TP53* mutation classifications

TP53 mutation status	N	(%)	HR	95% CI	P Value
Wild-type	140	42.0%	1.00		
DBD missense mutations	157	47.1%	1.78	1.23-2.57	0.002
All other mutations	36	10.8%	1.02	0.54-1.93	0.955
					
Wild-type	140	42.0%	1.00		
Evolutionary action score high	111	33.3%	1.99	1.35-2.93	0.001
Evolutionary action score low	49	14.7%	1.49	0.90-2.49	0.124
					
Wild-type	140	42.0%	1		
L2, L3, LSH	133	39.9%	1.78	1.21-2.59	0.003
Mutation outside of L2,L3,LSH	60	18.0%	1.31	0.80-2.16	0.276
					
Wild-type	140	42.0%	1.00		
Disruptive	81	24.3%	1.38	0.88-2.15	0.158
Nondisruptive	112	33.6%	1.82	1.23-2.69	0.003
					
Wild-type	140	42.0%			
Truncating	44	13.2%	1.45	0.85-2.48	0.177
Non-truncating	149	44.7%	1.68	1.16-2.43	0.007

P values were calculated with univariate Cox regression analysis.

We then compared the performance of our classification with that of other previously reported methods. Disruptive and truncating mutations were not associated with a significantly decreased DSS compared with patients with wild-type *TP53* (disruptive: HR, 1.38; 95% CI, 0.88−2.15; P=0.158, truncating: HR, 1.45; 95% CI, 0.85−2.48; P=0.177). In contrast, Evolutionary Action score (EAp53) high-risk mutations and mutations in the L2, L3 or LSH regions were good predictors of a shorter DSS compared with wild-type *TP53* (EAp53: HR, 1.99; 95% CI, 1.35−2.93; P=0.001, L2, L3, LSH: 1.78; 95% CI, 1.21−2.59; P=0.003). However, the DSS of patients harboring *TP53* mutations not included in these classifications was also reduced compared with wild-type *TP53*, albeit not significantly so (low-risk EAp53: HR, 1.49; 95% CI, 0.90−2.49; P=0.124, mutations outside of the L2, L3 or LSH regions: HR, 1.31; 95% CI, 0.80-2.16; P=0.276). Consequently, only DBD missense mutations allowed a clear identification of patients with a poor prognosis compared with wild-type *TP53*.

### *TP53* DBD missense mutations are associated with decreased DFS, DSS, and OS

Because *TP53* DBD missense mutations allowed the best discrimination between low- and high-risk *TP53* mutation subgroups, we performed a detailed analysis of their association with other survival endpoints (Figure 3B-3G). Similar to DSS, a carriage of *TP53* DBD missense mutations was associated with a significantly decreased DFS compared with wild-type *TP53* (HR, 1.38; 95% CI, 1.01−1.92; P=0.049; Figure 3B). In keeping with the results obtained for DSS, the DFS of patients bearing all other mutations was comparable to that of patients with wild-type *TP53* and better than that of patients with *TP53* DBD missense mutations. The difference between *TP53* DBD missense mutations and all other mutations was of borderline statistical significance for both DFS and DSS (DFS: HR, 1.57; 95% CI, 0.93−2.41; P=0.099, DSS HR, 1.75; 95% CI, 0.98−2.68; P=0.065). Similar to DFS and DSS, OS was lower for patients carrying *TP53* DBD missense mutations compared with wild-type *TP53* (HR, 1.64; 95% CI, 1.23−2.22; P=0.001). In patients with all other mutations, five cases of death occurring after a follow-up of more than 100 months led to a decreased OS. Because these deaths were unrelated to the primary tumor, we reasoned that second primary tumors might have been a contributing factor. The percentage of second primary tumors was nonsignificantly higher in patients with all other mutations compared with wild-type *TP53* (27.8% and 15.7%, respectively, P=0.143). A second primary tumor was observed in three of the five patients with all other mutations who died after >100 months of follow-up. Despite the decreased OS observed during the late follow-up period, the OS of patients with all other mutations and wild-type *TP53* did not differ significantly from each other (P=0.214).

Because of their similar survival characteristics (especially in terms of DFS and DSS), patients with all other mutations and wild-type *TP53* were grouped together for the purpose of analysis. Compared with the combined group, patients with *TP53* DBD missense mutations were found to have significantly shorter DFS, DSS, and OS (DFS: HR, 1.42; 95% CI, 1.06−1.97; P=0.022, DSS: HR, 1.78; 95% CI, 1.29−2.54; P=0.001; OS: HR, 1.55; 95% CI, 1.19-2.10; P=0.002; Figure 3E-3G). The distribution of the majority of risk factors was similar in patients with all other mutations and wild-type *TP53*, confirming that the two groups were biologically comparable. Compared with patients with *TP53* DBD missense mutations, those with all other mutations and wild-type *TP53* had a lower AJCC T-classification (P=0.036), AJCC overall stage (P=0.016), a lower risk of bone marrow invasion (P=0.014), and distant metastases (P<0.001; Table 3).

**Table 3:** General characteristics of patients according to the type of *TP53* mutations

Characteristics	TP53 Wt		TP53 All other mutations		TP53 Wt/All other mutations		TP53 DBD missense mutations		P value
	N	(%)	N	(%)	N	(%)	N	(%)	
**Entire study cohort**	140	(42.0%)	36	(10.8%)	176	(52.8%)	157	(47.1%)	
**Sex**									1.000
Male	129	(92.1%)	36	(100.0%)	165	(93.8%)	148	(94.3%)	
Female	11	(7.9%)	0	(0.0%)	11	(6.3%)	9	(5.7%)	
**Age, years**									
Median	47		47.5		47		49		
Range	29	−89	31	−71	29	−89	27	−83	
Mean ± SD	49.5	±11.7	48.9	±11.1	49.4	±11.5	50.1	±10.5	
**Age (categorical)**									0.389
< 65 years	120	(85.7%)	33	(91.7%)	153	(86.9%)	142	(90.4%)	
≥ 65 years	20	(14.3%)	3	(8.3%)	23	(13.1%)	15	(9.6%)	
**Cigarette smoking**									0.463
No	15	(10.7%)	4	(11.1%)	19	(10.8%)	13	(8.3%)	
Yes	125	(89.3%)	32	(88.9%)	157	(89.2%)	144	(91.7%)	
**Betel chewing**									0.261
No	30	(21.4%)	7	(19.4%)	37	(21.0%)	25	(15.9%)	
Yes	110	(78.6%)	29	(80.6%)	139	(79.0%)	132	(84.1%)	
**Alcohol drinking**									0.333
No	49	(35.0%)	6	(16.7%)	55	(31.3%)	41	(26.1%)	
Yes	91	(65.0%)	30	(83.3%)	121	(68.8%)	116	(73.9%)	
**HPV16/18 positive**									0.245
No	111	(79.3%)	29	(80.6%)	140	(79.5%)	135	(86.0%)	
Yes	20	(14.3%)	6	(16.7%)	26	(14.8%)	16	(10.2%)	
Unknown	9	(6.4%)	1	(2.8%)	10	(5.7%)	6	(3.8%)	
**Tumor site**									0.175
Buccal	61	(43.6%)	16	(44.4%)	77	(43.8%)	50	(31.8%)	
Retromolar Trigone	3	(2.1%)	1	(2.8%)	4	(2.3%)	11	(7.0%)	
Lip	1	(0.7%)	0	(0.0%)	1	(0.6%)	1	(0.6%)	
Tongue	46	(32.9%)	15	(41.7%)	61	(34.7%)	64	(40.8%)	
Alveolar ridge	21	(15.0%)	2	(5.6%)	23	(13.1%)	20	(12.7%)	
Hard palate	2	(1.4%)	0	(0.0%)	2	(1.1%)	4	(2.5%)	
Mouth floor	6	(4.3%)	2	(5.6%)	8	(4.5%)	7	(4.5%)	
**AJCC T-classification**									0.036
pT1-2	69	(49.3%)	19	(52.8%)	88	(50.0%)	60	(38.2%)	
pT3-4	71	(50.7%)	17	(47.2%)	88	(50.0%)	97	(61.8%)	
**AJCC N-classification**									0.068
pN1	55	(39.3%)	16	(44.4%)	71	(40.3%)	48	(30.6%)	
pN2	85	(60.7%)	20	(55.6%)	105	(59.7%)	109	(69.4%)	
**AJCC staging (overall)**									0.016
p-Stage III	41	(29.3%)	12	(33.3%)	53	(30.1%)	29	(18.5%)	
p-Stage IV	99	(70.7%)	24	(66.7%)	123	(69.9%)	128	(81.5%)	
**Extracapsular spread**									0.122
No	63	(45.0%)	18	(50.0%)	81	(46.0%)	59	(37.6%)	
Yes	77	(55.0%)	18	(50.0%)	95	(54.0%)	98	(62.4%)	
**Differentiation**									0.870
Well	24	(17.1%)	9	(25.0%)	33	(18.8%)	27	(17.2%)	
Moderate	93	(66.4%)	21	(58.3%)	114	(64.8%)	106	(67.5%)	
Poor	23	(16.4%)	6	(16.7%)	29	(16.5%)	24	(15.3%)	
**Bone marrow invasion**									0.014
No	116	(82.9%)	33	(91.7%)	149	(84.7%)	115	(73.2%)	
Yes	24	(17.1%)	3	(8.3%)	27	(15.3%)	42	(26.8%)	
**Skin invasion**									0.365
No	127	(90.7%)	34	(94.4%)	161	(91.5%)	138	(87.9%)	
Yes	13	(9.3%)	2	(5.6%)	15	(8.5%)	19	(12.1%)	
**Perineural invasion**									0.743
No	68	(48.6%)	16	(44.4%)	84	(47.7%)	78	(49.7%)	
Yes	72	(51.4%)	20	(55.6%)	92	(52.3%)	79	(50.3%)	
**Vascular invasion**									0.629
No	132	(94.3%)	33	(91.7%)	165	(93.8%)	150	(95.5%)	
Yes	8	(5.7%)	3	(8.3%)	11	(6.3%)	7	(4.5%)	
**Lymphatic invasion**									0.742
No	124	(88.6%)	31	(86.1%)	155	(88.1%)	136	(86.6%)	
Yes	16	(11.4%)	5	(13.9%)	21	(11.9%)	21	(13.4%)	
**Margin status**									0.065
< 5 mm	10	(7.1%)	6	(16.7%)	16	(9.1%)	25	(15.9%)	
≥ 5 mm	129	(92.1%)	30	(83.3%)	159	(90.3%)	129	(82.2%)	
Unknown	1	(0.7%)	0	(0.0%)	1	(0.6%)	3	(1.9%)	
**Tumor depth**									0.244
< 10 mm	53	(37.9%)	11	(30.6%)	64	(36.4%)	47	(29.9%)	
≥ 10 mm	86	(61.4%)	25	(69.4%)	111	(63.1%)	110	(70.1%)	
Unknown	1	(0.7%)	0	(0.0%)	1	(0.6%)	0	(0.0%)	
**Local recurrence**									1.000
No	111	(79.3%)	30	(83.3%)	141	(80.1%)	126	(80.3%)	
Yes	29	(20.7%)	6	(16.7%)	35	(19.9%)	31	(19.7%)	
**Neck recurrence**									0.699
No	108	(77.1%)	28	(77.8%)	136	(77.3%)	118	(75.2%)	
Yes	32	(22.9%)	8	(22.2%)	40	(22.7%)	39	(24.8%)	
**Distant metastasis**									<0.001
No	118	(84.3%)	30	(83.3%)	148	(84.1%)	104	(66.2%)	
Yes	22	(15.7%)	6	(16.7%)	28	(15.9%)	53	(33.8%)	
**Level IV/V metastases**									0.214
No	132	(94.3%)	34	(94.4%)	166	(94.3%)	142	(90.4%)	
Yes	8	(5.7%)	2	(5.6%)	10	(5.7%)	15	(9.6%)	
**Second primary tumor**									0.780
No	118	(84.3%)	26	(72.2%)	144	(81.8%)	126	(80.3%)	
Yes	22	(15.7%)	10	(27.8%)	32	(18.2%)	31	(19.7%)	
**Relapse after complete treatment**									0.037
No	79	(56.4%)	21	(58.3%)	100	(56.8%)	71	(45.2%)	
Yes	61	(43.6%)	15	(41.7%)	76	(43.2%)	86	(54.8%)	
**OSCC-related death**									0.001
No	95	(67.9%)	24	(66.7%)	119	(67.6%)	78	(49.7%)	
Yes	45	(32.1%)	12	(33.3%)	57	(32.4%)	79	(50.3%)	
**Death from any cause**									0.010
No	71	(50.7%)	12	(33.3)	83	(47.2%)	52	(33.1%)	
Yes	69	(49.3%)	24	(66.7)	93	(52.8%)	105	(66.9%)	

Characteristics of patients with wild-type *TP53*, all other *TP53* mutations, all other *TP53* mutations combined with wild-type *TP53*, and *TP53* DBD missense mutations. Categorical data (patients with wild-type *TP53* combined with all other mutations *versus* patients with DBD missense mutations) were compared with the Fisher's exact test or the χ^2^ test, as appropriate.

### *TP53* DBD missense mutations are an independent prognostic factor for reduced DSS

We next sought to identify the prognostic factors for DSS (Table 4). In univariate analysis, we identified advanced AJCC T-classification, N-classification, and overall stage, ECS, and *TP53* DBD missense mutations as the main risk factors for DSS (P≤0.001). Other factors included tumor differentiation, invasion to bone marrow, skin, and lymphatic vessels, as well as margin status, tumor depth, and the occurrence of any *TP53* mutation. After allowance for potential confounders, multivariate analysis revealed that *TP53* DBD missense mutations retained their independent prognostic significance for DSS (HR, 1.55; 95% CI, 1.09−2.20; P=0.014). Other independent predictors were advanced AJCC T-classification (HR, 1.94; 95% CI, 1.33−2.81; P=0.001), N-classification (HR, 1.55; 95% CI, 1.01−2.38; P=0.047), and the presence of ECS (HR, 1.69; 95% CI, 1.12−2.55; P=0.013).

**Table 4:** Univariate and multivariate analyses of risk factors in relation to disease-specific survival

Variable	N	(%)	HR^(a)^	95% CI^(a)^	P Value^(a)^	HR^(b)^	95% CI^(b)^	P Value^(b)^
**Risk factor**								
**Sex (Male vs Female)**	313	(94.0%)	1.73	0.71-4.22	0.230			
**Age (≥65 years vs <65 years)**	38	(11.4%)	0.88	0.51-1.53	0.649			
**HPV status (16/18 positive vs HPV16/18 negative)**	42	(12.6%)	1.05	0.64-1.70	0.860			
**AJCC T-classification (pT3-4 vs pT1-2)**	185	(55.6%)	2.31	1.60-3.33	<0.001	1.94	1.33-2.81	0.001
**AJCC N-classification (pN2 vs pN1)**	214	(64.3%)	2.22	1.50-3.29	<0.001	1.55	1.01-2.38	0.047
**AJCC staging (IV vs III)**	251	(75.4%)	2.61	1.61-4.25	<0.001			
**Extracapsular spread (Yes vs No)**	193	(58.0%)	2.37	1.63-3.44	<0.001	1.69	1.12-2.55	0.013
**Differentiation (Poor vs Well/Moderate)**	53	(15.9%)	1.65	1.09-2.51	0.018			
**Bone marrow invasion (Yes vs No)**	69	(20.7%)	1.50	1.02-2.20	0.040			
**Skin invasion (Yes vs No)**	34	(10.2%)	1.69	1.05-2.72	0.030			
**Perineural invasion (Yes vs No)**	171	(51.4%)	1.27	0.91-1.79	0.160			
**Vascular invasion (Yes vs No)**	18	(5.4%)	0.95	0.44-2.03	0.892			
**Lymphatic invasion (Yes vs No)**	42	(12.6%)	1.85	1.20-2.85	0.006			
**Margin status (<5 mm vs ≥5 mm)**	41	(12.3%)	1.91	1.22-2.99	0.005			
**Tumor depth (≥10 mm vs <10 mm)**	221	(66.4%)	1.76	1.20-2.60	0.004			
**TP53 Mutation**								
**Mutant vs Wildtype**	193	(58.0%)	1.62	1.14-2.32	0.008			
**DBD missense mutations vs Wt/All other mutations**	157	(47.1%)	1.78	1.26-2.50	0.001	1.55	1.09-2.20	0.014

P values were calculated with Cox regression using a forward selection procedure for multivariate analysis.

a Univariate analysis

b Multivariate analysis

### *TP53* DBD missense mutations combined with traditional clinical risk factors identify high-risk OSCC patients

**Figure 4:**
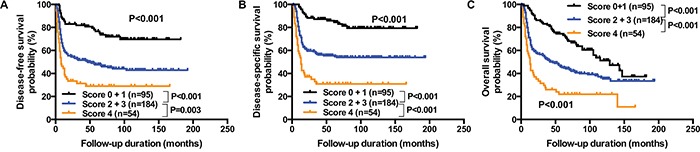
Prognostic scoring system for DSS The scoring system is based on the four independent predictors of DSS identified by multivariate analysis (i.e., presence of a *TP53* DBD missense mutation, ECS, advanced AJCC N- and T-classification). One point was attributed to each risk factor present. Three risk categories were identified, as follows: low-risk (scores of 0−1; n=95, 28.5%), intermediate-risk (scores of 2−3; n=184, 55.3%) and high-risk (score of 4; n=54, 16.2%). Kaplan-Meier plots for DFS **panel A.**, DSS **panel B.**, and OS **panel C.** for the three risk groups were constructed. P values were calculated with the log-rank test.

We finally reasoned that the difference in DSS between patients bearing *TP53* DBD missense mutations and patients with all other mutations or wild-type *TP53* was less than 20% (49.7% *versus* 67.6%, respectively). Furthermore, the high prevalence of *TP53* DBD missense mutations (47.1%) prevented a clear identification of high-risk patients. In an effort to improve patient stratification, we devised a prognostic scoring system based on the four independent predictors of DSS identified on multivariate analysis. One point was attributed to each risk factor present. Three risk categories were identified, as follows: low-risk (scores of 0−1; n=95, 28.5%), intermediate-risk (scores of 2−3; n=184, 55.3%) and high-risk (score of 4; n=54, 16.2%) (Table 5 and Figure 4).

**Table 5:** Prognostic scoring system for OSCC patient survival

Score	N	(%)	DFS	HR	95% CI	P Value	DSS	HR	95% CI	P Value	OS	HR	95% CI	P Value
**0 + 1**	95	(28.5%)	73.7%				82.1%				56.8%			
**2 + 3**	184	(55.3%)	46.2%	2.64	1.70-4.10	< 0.001	55.4%	3.22	1.91-5.44	< 0.001	38.0%	1.90	1.33-2.72	< 0.001
**4**	54	(16.2%)	29.6%	4.60	2.76-7.68	< 0.001	31.5%	6.78	3.79-12.14	< 0.001	20.4%	3.92	2.53-6.06	< 0.001

The scoring system is based on the four independent risk factors identified by multivariate analysis (i.e., presence of a *TP53* DBD missense mutation, ECS, advanced AJCC N- and T-classifications). P values were calculated with Cox regression using patients with scores of 0−1 as the reference category.

DFS, DSS, and OS of intermediate-risk and high-risk patients were significantly lower than those observed in low-risk patients (all P<0.001). Specifically, the hazard ratios for DFS, DSS, and OS were 2.64, 3.22, and 1.90 for intermediate-risk patients, and 4.60, 6.78 and, 3.92 for high-risk patients, respectively. The DFS rates of low-, intermediate-, and high-risk patients were 73.7%, 46.2%, and 29.6%, respectively; DSS rates were 82.1%, 55.4%, and 31.5%, and OS rates were 56.8%, 38.0%, and 20.4%, respectively.

## DISCUSSION

Interest into the prognostic significance of traditional [3, 20] and genetic [19, 21] risk factors in OSCC is mounting. Previous studies have shown that *TP53* expression [22] and mutation status [6, 7] can predict prognosis in OSCC patients. In the present study, we demonstrate that *TP53* DBD missense mutations were the optimal classifier for distinguishing between *TP53* mutant patients with low and high clinical risk. We also show that *TP53* DBD missense mutations can be used in combination with traditional risk factors for improving prognostic stratification.

With regard to *TP53* mutation subtypes, we were unable to confirm a particularly deleterious effect of truncating mutations [9]. Truncating mutations are included in the group of disruptive mutations [8], which may likely have contributed to the unsatisfactory results obtained with this classification method in our cohort. In contrast, mutations in L2, L3 or LSH [7, 16, 23] and high-risk missense mutations according to the EAp53 [14, 15] were capable of identifying high-risk patients in a successful manner. We believe that this capacity may be attributed to the fact that they include a high proportion of a gain-of-function mutations [10]. However, these classification approaches were inferior to *TP53* DBD missense mutations for distinguishing between high- and low-risk *TP53* mutation carriers. It is feasible that this could be attributed to their lack of inclusion of all missense mutations occurring in the DBD that mediate an enhanced risk. It should be noted that EAp53 also includes missense mutations located outside of DBD. However, the vast majority of missense mutations are located within the DBD, indicating a selection for missense mutations in this region. The low number of missense mutations located outside of the DBD complicates the assessment of their prognostic impact. However, amino acid substitutions occurring outside of the DBD may have biological effects. For example, an arginine variant located at codon 72 is associated with a higher likelihood of apoptosis [24] and a later disease onset in its carriers [25].

The results of our study indicate that *TP53* DBD missense mutations − but not all of the remaining mutations (defined as “all other mutations” in this study) − are significantly associated with reduced DFS, DSS, and OS in patients with advanced OSCC. However, the survival difference between patients with *TP53* DBD missense mutations and all other mutations did not reach statistical significance (P=0.099 and P=0.065 for DFS and DSS, respectively), most likely because of the small sample size (n = 36) of patients harboring all other mutations. We also observed a decrease in OS for patients with all other mutations during the late follow-up period. Although this phenomenon was unrelated to primary disease, there was a tendency toward an increased incidence of second primary tumors in patients with all other mutations compared with wild-type *TP53* (P=0.143). Future studies are required to investigate whether the observed late reduction in OS is a hallmark of patients with all other mutations and to indicate whether these patients may need a closer follow-up schedule.

It is noteworthy that nearly 50% of all patients included in the current study were carriers of *TP53* DBD missense mutations. In addition, the difference in terms of DSS between these patients and patients with wild-type *TP53* or all other mutations was less than 20%. This hampered the identification of a specific subgroup of patients at high clinical risk. The analysis of *TP53* mutations in combination with other risk factors (e.g., nodal status or 3p loss) was shown to be clinically useful for predicting treatment outcomes and survival [26, 27]. We therefore devised a prognostic scoring system that combined the presence of *TP53* DBD missense mutations with traditional prognostic factors. The combination of *TP53* DBD missense mutations with the three independent prognostic factors for DSS identified in multivariate analysis (AJCC N-classification, AJCC T-classification, and ECS) allowed the identification of three distinct risk groups, i.e., low-risk (28.5% of the study patients), intermediate-risk (55.3%), and high-risk (16.2%) patients. We believe that such stratification can rationalize both OSCC treatment and clinical follow-up.

Notably, *TP53* mutations have been associated with a decreased response to both 5-fluorouracil [26] and radiotherapy [16]. Additionally, *TP53* mutation subtypes may directly guide future targeted treatment. Viral therapy may be considered for loss-of-function mutations, whereas small molecules targeting gain-of-function mutations may be used to restore *TP53* functionality [28]. Small molecules have entered human clinical trials [29], and viral therapy has shown promising results in patients with head and neck malignancies [30, 31].

Some caveats of our study merit comment. First, all of the study patients were Taiwanese and the research was conducted in a betel nut chewing endemic area. In this regard, ethnicity and different combinations of environmental factors [32] – including exposure to agents other than tobacco and alcohol [33] – have been shown to influence the occurrence or the spectrum of *TP53* mutations. The question as to whether our findings are generalizable to other ethnic groups deserves further scrutiny. Consequently, our scoring system should be externally validated in independent cohorts. Second, only patients with advanced carcinoma were included. It is noteworthy that *TP53* DBD missense mutations were associated with a higher prevalence of AJCC stage IV disease as compared with stage III. Additional investigations on the potential prognostic effects of *TP53* DBD missense mutations in patients with earlier stages of disease would be desirable. We recognize that a complete sequencing of all exons would have led to the identification of a higher number of patients with both *TP53* DBD missense mutations and other mutations. In this regard, *TP53* mutations at positions not covered in this study have been reported to occur in HNSCC patients [4]. Finally, our study is limited by its retrospective nature. Further research with a longitudinal design is warranted to confirm and expand our data.

In conclusion, our results demonstrate that *TP53* DBD missense mutations are an independent adverse prognostic factor in patients with advanced OSCC and may improve risk stratification when combined with traditional clinicopathological parameters. Future studies are necessary to clarify whether this prognostic tool can rationalize both OSCC treatment and clinical follow-up.

## MATERIALS AND METHODS

### Samples

Tumor samples were collected from 345 pathological node-positive patients with AJCC stage III or IV OSCC who were referred to the Chang Gung Memorial Hospital between 1996 and 2009. All patients were treated with radical surgery either with or without subsequent adjuvant radiotherapy/concurrent chemoradiotherapy. The study protocol complied with the tenets of the Helsinki declaration and was approved by the Institutional Review Board of the Chang Gung Memorial Hospital (CGMH 101-4457B). Because of the retrospective nature of the study, the need for patient consent was waived.

### Mutation analysis of *TP53*

The mutation analysis of *TP53* has been previously published as part of a large genomic OSCC study [19] and the samples analyzed in the current study are the same reported previously. However, the association of different *TP53* mutations with survival was not specifically analyzed. Genomic DNA was extracted from FFPE samples with the QIAamp DNA FFPE Tissue Kit (Qiagen, Hilden, Germany) according to the manufacturer's instructions. The Quant-iT™ dsDNA HS Assay (Invitrogen, Carlsbad, CA, USA) was used for quantification of isolated DNA. The generation of target amplicon libraries was performed with the Ion AmpliSeq™ cancer panel primer pool and Ion AmpliSeq kit 2.0 (Applied Biosystems, Foster City, CA, USA) according to the manufacturer's protocol. Genomic DNA (20 ng) served as a template for multiplex PCR. The *TP53* amplicons covered 51% of the 393 aminoacids (including exons 2, 4−8 and 10) as previously described [19]. PCR reactions were followed by ligation to barcode adapters and five amplification cycles. The libraries were used for emulsion PCR (emPCR) amplification using Ion Sphere™ particles on an Ion OneTouch System (Applied Biosystems). Samples were sequenced on an Ion 318 Chip (Applied Biosystems) using the Ion Personal Genome Machine (PGM) following the manufacturer's instructions. An alignment with the hg19 reference genome was performed for data analysis, followed by the identification of genetic variants. To this aim, the Ion Torrent Suite software (v. 3.2) and the Torrent Variant Caller software (v. 3.2) were used.

### Mutation classification

We annotated all variants located in exons with an allelic count ≥ 25× and an allelic frequency ≥ 5%. Annotation was performed with the ANNOVAR and Cancer panel analysis pipeline (CPAP). Common single nucleotide polymorphisms without known clinical relevance were identified with the dbSNP138 database and subsequently disregarded. Only non-synonymous mutations were included in further analyses. The following *TP53* mutation classifiers were analyzed in relation to their prognostic impact: 1) *TP53* DBD missense mutations; 2) high-risk EAp53 mutations; 3) mutations in the L2, L3 or LSH regions; 4) disruptive mutations; and 5) truncating mutations. When multiple mutations were identified in the same patient, the presence of at least one *TP53* mutation deemed to be deleterious for the corresponding classifier was sufficient for considering the subject as a mutation carrier. The classifiers were defined as follows: 1) “*TP53* DBD missense mutations” were defined as missense mutations the residues 95−289, whereas all of the remaining mutations were defined as “all other mutations”; 2) mutations were classified as EAp53 high-risk and low-risk according to a previously published methodology [14]; 3) mutations in the L2, L3 or LSH regions were defined as mutations of the residues 164−194 (L2), 237−250 (L3), and 119−135 or 272−287 (LSH); 4) disruptive mutations were identified as previously described [8], and 5) all frameshift, nonsense, and splice-site mutations were considered as “truncating”.

### Statistical analysis

Categorical data were compared with the Fisher's exact test (2 × 2 contingency tables) or the χ^2^ test, as appropriate. Disease-free survival (DFS), disease-specific survival (DSS), and overall survival (OS) curves were plotted with the Kaplan-Meier method and compared with the log-rank test. DFS was defined as the time between surgery and TNM stage recurrence or the date of the last follow-up. DSS was calculated as the time from surgery to the date of death related to primary OSCC or the last follow-up. OS was defined as the time between surgery and death from any cause or the last follow-up. Univariate and multivariate analyses of DSS were based on Cox regression models. The following 17 clinicopathological variables were included in the analysis: age, sex, HPV16/18 infections, pathological AJCC T-classification, N-classification, and overall stage, ECS, differentiation, invasion to bone marrow, skin, nerve, blood vessel or lymphatic vessel, pathological margin status, tumor depth, presence of any *TP53* mutation, and presence of a *TP53* DBD missense mutation. The forward selection method was applied for multivariate analysis. Results were expressed as hazard ratios (HRs) with 95% confidence intervals (CIs). We then devised a prognostic scoring system based on the four independent predictors of DSS identified on multivariate analysis (i.e., presence of a *TP53* DBD missense mutation, ECS, advanced AJCC N-classification, and advanced AJCC T-classification). One point was attributed to each risk factor present. DFS and DSS curves of low-risk (scores of 0−1), intermediate-risk (scores of 2−3) and high-risk (score of 4) patients were summarized with the Kaplan-Meier method and compared using the log-rank test. All calculations were performed with the GraphPad Prism (v. 6.0; GraphPad Inc., San Diego, CA, USA) and SPSS (v. 20.0.0; IBM, Somers, NY, USA) statistical packages.

## SUPPLEMENTARY TABLES








