# Supplementary material for: Missense mutations in the TP53 DNA-binding domain predict outcomes in patients with advanced oral cavity squamous cell carcinoma
Source: Oncotarget. 2016 Jun 8;7(28):44194–210. doi: 10.18632/oncotarget.9925 (PMC5190089; doi:10.18632/oncotarget.9925)
Supplement: Supplementary file 1 [file oncotarget-07-44194-s001.pdf]

Missense mutations in the *TP53* DNA-binding domain predict outcomes in patients with advanced oral cavity squamous cell carcinoma

SUPPLEMENTARY TABLES

Supplementary Table S1: TP53 mutations and their frequencies at different amino acid positions in a total of 193 tumor specimens (total number of patients: 333)

See Supplementary File 1

Supplementary Table S2: Complete list of all 228 TP53 mutations detected in a total of 193 tumor specimens (total number of patients: 333)

See Supplementary File 2

Supplementary Table S3: Characteristics of all study participants (n=333)

See Supplementary File 3
